# The association between depression and metabolic syndrome and its components: a bidirectional two-sample Mendelian randomization study

**DOI:** 10.1038/s41398-021-01759-z

**Published:** 2021-12-13

**Authors:** Min Zhang, Jing Chen, Zhiqun Yin, Lanbing Wang, Lihua Peng

**Affiliations:** 1 School of Public Health and Management, Chongqing Medical University, Chongqing, 400016 China; 2 Department of Anesthesia and Pain Medicine, The First Affiliated Hospital of Chongqing Medical University, Chongqing, 400016 China; 3 Department of Psychiatry and Psychology, No.964 Hospital of People’s Liberation Army, Changchun City, 130026 Jilin Province China; 4 Division of medical affairs, The First Affiliated Hospital of Army Military Medical University, Chongqing, 400038 China

**Keywords:** Psychology, Depression, Medical genetics

## Abstract

Observational studies suggested a bidirectional correlation between depression and metabolic syndrome (MetS) and its components. However, the causal associations between them remained unclear. We aimed to investigate whether genetically predicted depression is related to the risk of MetS and its components, and vice versa. We performed a bidirectional two-sample Mendelian randomization (MR) study using summary-level data from the most comprehensive genome-wide association studies (GWAS) of depression (*n* = 2,113,907), MetS (*n* = 291,107), waist circumference (*n* = 462,166), hypertension (*n* = 463,010) fasting blood glucose (FBG, *n* = 281,416), triglycerides (*n* = 441,016), high-density lipoprotein cholesterol (HDL-C, *n* = 403,943). The random-effects inverse-variance weighted (IVW) method was applied as the primary method. The results identified that genetically predicted depression was significantly positive associated with risk of MetS (OR: 1.224, 95% CI: 1.091–1.374, *p* = 5.58 × 10^−4^), waist circumference (OR: 1.083, 95% CI: 1.027–1.143, *p* = 0.003), hypertension (OR: 1.028, 95% CI: 1.016–1.039, *p* = 1.34 × 10^−6^) and triglycerides (OR: 1.111, 95% CI: 1.060–1.163, *p* = 9.35 × 10^−6^) while negative associated with HDL-C (OR: 0.932, 95% CI: 0.885–0.981, *p* = 0.007) but not FBG (OR: 1.010, 95% CI: 0.986–1.034, *p* = 1.34). No causal relationships were identified for MetS and its components on depression risk. The present MR analysis strength the evidence that depression is a risk factor for MetS and its components (waist circumference, hypertension, FBG, triglycerides, and HDL-C). Early diagnosis and prevention of depression are crucial in the management of MetS and its components.

## Introduction

Depression is a common mental health illness. The lifetime prevalence of depression varies across countries with a midpoint of about 10.0% and is generally higher in higher-income than lower-income countries [1–3]. Globally, depression is estimated to be one of the leading causes of “years lost” to a disability, resulting in a loss of individual productivity, placing a severe economic burden [4–6]. In its most severe cases, depression can lead to suicide [7–9], contributing to the increased risk of mortality [10]. With the increasing incidence [11], depression is one of the most challenging public health issues today.

Another big health threat is metabolic syndrome (MetS), which is defined as a clustering of abdominal obesity, hyperglycemia, elevated blood pressure, and dyslipidemia [12]. The prevalence of MetS varies in different ethnicity due to the criteria used for MetS definitions and has steadily risen worldwide [13, 14]. It was estimated that MetS has affected over a billion people globally [13]. MetS and its components are associated with various diseases and overall mortality, making it a severe health problem and economic burden [15, 16].

As both depression and MetS are risk factors for cardiovascular disease [15, 17], their relationship has been attracted extensive attention in recent years [18, 19]. Epidemiological data have consistently indicated a co-occurrence of depression with MetS and its components [20–22]. Several meta-analyses and reviews also suggested a bidirectional relationship between depression and MetS [23–26]. It has been shown that depression increased MetS risk by 34% in cross-sectional studies and 52% in cohort studies. Conversely, patients with MetS had a 1.27-fold (cross-sectional studies) to 1.49 times (cohort studies) higher risk of depression than controls [23]. However, the bidirectional correlations between depression and MetS and its components identified in observational studies might be susceptible to confounding factors, small sample size, limited follow-up time, and reverse causation, which might mislead the conclusion [27]. Thus, the potential causality of depression in determining the risk of MetS and its components remains elusive and vice versa.

Mendelian randomization (MR) is a more robust method of causal inferences, which could overcome the limitations of observational studies [28, 29]. MR uses genetic variations that are robustly associated with exposure as an instrument, which might effectively avoid the influence of confoundings and reverse causes [28, 30–32]. Genome-wide association studies (GWAS) have identified thousands of variants related to complex exposures, which has pushed the widespread use of MR to a new climax [33, 34]. In this study, we utilized a bidirectional two-sample MR method to examine bidirectional causality between depression and MetS and its components.

## Materials and methods

### Study design overview

**Fig. 1:**
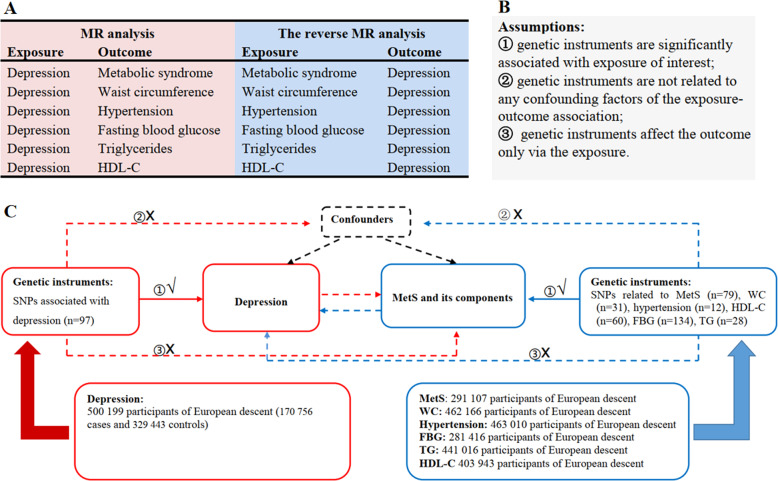
Overview of the study design in this bidirectional MR study. **A** We performed a total of 12 MR analyses to investigate the bidirectional association between depression and MetS and its components. **B** MR analysis depends on three key assumptions. **C** Sketch of the study design. MetS metabolic syndrome, FBG fasting blood glucose, TG triglycerides, WC waist circumference, HDL-C high-density lipoprotein cholesterol.

A brief description of this bidirectional MR design is shown in Fig. 1. The components of MetS were charactered with five elements according to the National Cholesterol Education Program Adult Treatment Panel III (NCEP/ATP III) criteria [35]. We tested the causal relationship of depression on MetS and its components. We adopted the summary-level statistics from the most comprehensive meta-analyses of GWASs for MetS, hypertension, waist circumference, fasting blood glucose (FBG), serum triglycerides (TG), and serum high-density lipoprotein cholesterol (HDL-C). In the reverse-direction MR analysis, we assessed the association between genetically predicted MetS and its components and depression risk. Summary-level data from the most extensive meta-analyses of GWASs for depression was also extracted. Thus, we performed a total of 12 MR analyses to investigate the bidirectional association between depression and metabolic syndrome and its components. MR depends on three key assumptions (Fig. 1): ① genetic instruments are significantly associated with exposure of interest; ② genetic instruments are not related to any confounding factors of the exposure-outcome association; ③ genetic instruments affect the outcome only via the exposure [36]. Details of the data sources used in this study are summarized in Supplementary Table 1. To minimize racial mismatches, our analyses are restricted to most of the participants of European descent.

### Selection of genetic instruments for MR analyses

For each exposure factor, the SNPs were filtered according to the three main assumptions of MR. Firstly, we included SNPs at a threshold of genome-wide significance (*p* < 5 × 10^−8^). Then, we retained variants with the lowest *p* value as independent instruments based on linkage disequilibrium (LD) as measured by *r*^2^ (when *r*^2^ > 0.1 in the European 1000 Genome reference panel). Finally, to quantify the strength of instrumental variables, we calculated *F*-statistics, and a threshold of the *F*-statistics >10 was typically recommended for MR analyses.

### Data sources and SNP selection for depression

We used the hitherto largest published GWAS for depression, which included participants from 23andMe, Psychiatric Genomics Consortium (PGC), and UK Biobank with a total of 2,113,907 subjects (660,418 cases and 1,453,489 controls) [37]. Totally, 97 independent genetic SNPs were identified with genome-wide significant levels (*p* < 5 × 10^−8^) and were selected as genetic instruments for depression. The reverse-direction MR analyses used the summary-level data from PGC and UK Biobank with 500,199 individuals, which were publicly available (Supplementary Table 1).

### Data sources and SNP selection for MetS

Summary-level data for MetS were taken from the most comprehensive GWAS in UK Biobank [38], consisting of 291,107 individuals (59,677 cases and 231,430 controls) with nonmissing data on genotypes, outcomes, and covariates. For reverse MR analyses, 79 independent loci associated with MetS were identified in this GWAS with a genome-wide threshold of significance (*p* < 5 × 10^−8^), and we selected the satisfying variants for the construction of the instrumental variables (Supplementary Table 1).

### Data sources and SNP selection for the components of MetS

For waist circumference, we extracted the GWAS summary data from the Medical Research Council Integrative Epidemiology Unit (MRC-IEU) UK Biobank GWAS Pipeline, which included 462,166 subjects with European ancestry [39]. In the reverse-direction MR analyses, we selected 31 variants with a *p* value less than 5 × 10^−8^ recognized from a GWAS [40], which included up to 224,459 individuals from the Genetic Investigation of ANthropometric Traits (GIANT) Consortium for the construction of genetic instruments for waist circumference (Supplementary Table 1).

As to hypertension, the summary statistics were also available from the MRC-IEU UK Biobank pipeline, which included 463,010 subjects [39]. Twelve variants associated with hypertension at a genome-wide significance were identified from a GWAS of 29 studies [41] with 203,006 participants (GWAS: 69,395, and follow-up: 133,611) of European descent, and we selected those SNPs as the genetic instruments for hypertension (Supplementary Table 1).

For FBG, we obtained the summary-level data from the most comprehensive GWAS in the meta-analyses of glucose and insulin-related traits Consortium (MAGIC), which included 281,416 individuals with over 70% were European ancestry and were publicly available data [42]. A total of 134 SNPs were identified to be significantly associated with FBG (*p* < 5 × 10^−8^) and were selected as instruments for FBG (Supplementary Table 1).

For TG and HDL-C, the summary-level statistics were extracted from the UK Biobank with above 400,000 participants [43]. For the reverse-direction MR analyses, the variants were extracted from a most representative GWAS of 188,577 subjects from the Global Lipids Genetics Consortium (GLGC) [44]. This GWAS identified 28 and 60 SNPs significantly associated with TG and HDL-C (*p* < 5 × 10^−8^), respectively, and were used as instrumental variables (Supplementary Table 1).

### Statistical analyses

In this study, *R*^2^ was calculated to represent the proportion of variance in an exposure factor explained by the instrumental variables. *F*-statistic was calculated to represent the strength of the association between the instruments and risk of exposure of interest [45]. For binary exposures, the casual estimate was presented as an odds ratio (OR) and 95% confidence interval (CI) per log-odds increment in genetically determined risk of the exposures. As to continuous exposures, the causal estimate was presented as an OR with a 95% CI per standard deviation (SD) increment in an exposure. MR analyses utilized the random-effects inverse-variance weighted (IVW) method as the primary method to estimate the potential bidirectional causal associations between depression and MetS and its components as it provides a robust causal estimate in the absence of directional pleiotropy. Furthermore, we used weighted median, simple mode, weight mode, and MR-Egger methods for alternative analyses. Then, we performed tests for directional horizontal pleiotropy by MR-Egger intercept. We also tested the heterogeneity for MR-Egger regression and IVW method via Cochran’s *Q* statistics and funnel plots [46, 47]. In addition, sensitivity analysis by applying leave-one-out analysis was conducted. Post hoc power calculation for MR was based on online web tools (https://sb452.shinyapps.io/power/) [48]. All statistical analyses were performed using the TwoSampleMR packages in R (version 3.6.3, www.r-project.org/) or Stata 16 (Stata, College Station, TX). All *p* values are two-tailed. We adopted Bonferroni-corrected *p* value < 0.004 (0.05/12 = 0.004) to determine statistical significance in the MR analysis, and a *p* value less than 0.10 was considered significant in the MR-Egger test and heterogeneity test.

## Results

### The causal effect of depression on MetS and its components

Among the 97 depression-associated variants, two SNPs were not available in the summary-level datasets of wais circumference, hypertension, and triglycerides, and three SNPs were unavailable for HDL-C datasets. In addition, due to ambiguous palindrome, we excluded one variant for MetS and FBG, six variants for waist circumference and hypertension, five SNPs for triglycerides, and four SNPs for HDL-C. Thus, we finally included 96, 89, 89, 96, 90, and 90 variants as genetic instruments for MetS, waist circumference, hypertension, FBG, triglycerides, and HDL-C in the MR analyses, respectively. The *R*^2^ and *F*-statistics indicated that all genetic instruments were suitable for MR analysis, and most of the statistical power to test an OR of 1.20 was higher than 80.0% (Table 1).

**Table 1:** The *R*^2^ and *F*-statistics for the genetic instruments and the power for MR.

Exposure	Outcome	No. SNP	*R*^*2*^	*F*-statistic	Power
Depression	Metabolic syndrome	96	0.37%	77.71	99.6%
Depression	Waist circumference	89	0.33%	78.75	90.9%
Depression	Hypertension	89	0.32%	77.19	90.4%
Depression	Fasting blood glucose	96	0.32%	77.99	73.2%
Depression	Triglycerides	90	0.33%	78.47	89.8%
Depression	HDL-C	90	0.33%	77.00	86.5%
The reverse MR analysis					
Metabolic syndrome	Depression	73	2.34%	88.13	99.9%
Waist circumference	Depression	30	0.76%	55.57	100.0%
Hypertension	Depression	10	0.22%	37.21	100.0%
Fasting blood glucose	Depression	118	13.56%	230.46	100.0%
Triglycerides	Depression	26	2.84%	196.14	100.0%
HDL-C	Depression	60	3.32%	107.75	100.0%

*HDL-C* high-density lipoprotein cholesterol.

**Fig. 2:**
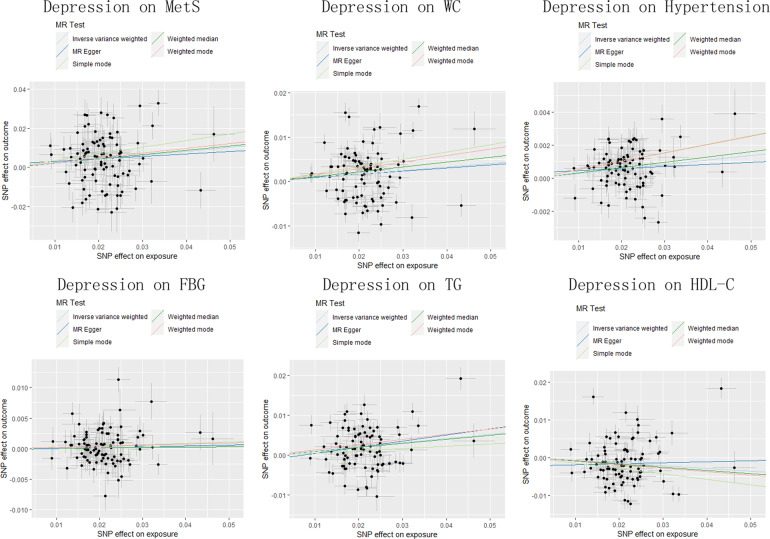
The scatter plots of the association between genetically predicted depression on MetS and its components. MetS metabolic syndrome, FBG fasting blood glucose, TG triglycerides, WC waist circumference, HDL-C high-density lipoprotein cholesterol.

The results of the MR analyses were shown in Table 2, the scatter plots and forest plots were presented in Fig. 2 and Supplementary Fig. 1, respectively. Genetically predicted depression was significantly positively associated with MetS. The OR with 95% CI of per log_-_odds increment in depression liability was 1.224 (95% CI: 1.091–1.374; *p* = 5.58 × 10^−4^) in the IVW model, which was consistent with the result of the weight median model. MR-Egger regression analysis showed no indication of potential non-horizontal pleiotropy (egger_intercept = 0.002, *p* = 0.747). The Cochran’s Q value suggested an obvious heterogeneity (Q = 271.41, *p* < 0.001) obtained from individual variants, but the funnel plot (Supplementary Fig. 2) showed an invisible asymmetry of the MR analyses, indicating no evidence of heterogeneity. Furthermore, the leave-one-out analysis suggested that the observed association was not significantly changed after removing any single variant (Supplementary Fig. 3).

**Table 2:** Genetic predicted depression on risk of MetS and its components in the MR analysis.

Exposure	Outcome	No.SNP	Methods	OR (95% CI)	*p* val	Egger_intercept	*p*-Egger_intercept
Depression	MetS	96	MR Egger	1.136 (0.711–1.814)	0.595	0.002	0.747
			IVW (Q = 271.41, *p* < 0.001)	1.224 (1.091–1.374)	5.59 × 10^−4^		
			Weighted median	1.244 (1.099–1.407)	5.22 × 10^−4^		
			Simple mode	1.410 (0.986–2.015)	0.063		
			Weighted mode	1.271 (0.880–1.836)	0.204		
Depression	Waist circumference	89	MR Egger	1.074 (0.863–1.335)	0.525	0.000	0.933
			IVW (Q = 691.11, *p* < 0.001)	1.083 (1.027–1.143)	0.003		
			Weighted median	1.115 (1.071–1.161)	1.15 × 10^−7^		
			Simple mode	1.181 (1.086–1.285)	1.99 × 10^−4^		
			Weighted mode	1.158 (1.067–1.256)	7.06 × 10^−4^		
Depression	Hypertension	89	MR Egger	1.013 (0.968–1.059)	0.587	0.000	0.507
			IVW (Q = 213.70, *p* < 0.001)	1.028 (1.016–1.039)	1.34 × 10^−6^		
			Weighted median	1.032 (1.020–1.045)	1.76 × 10^−7^		
			Simple mode	1.053 (1.020–1.086)	0.002		
			Weighted mode	1.052 (1.019–1.086)	0.002		
Depression	FBG	96	MR Egger	1.015 (0.921–1.119)	0.764	−0.000	0.912
			IVW (Q = 146.38, *p* < 0.001)	1.010 (0.986–1.034)	0.423		
			Weighted median	1.006 (0.977–1.036)	0.697		
			Simple mode	1.022 (0.942–1.109)	0.597		
			Weighted mode	1.018 (0.946–1.095)	0.634		
Depression	Triglycerides	90	MR Egger	1.168 (0.970–1.406)	0.106	−0.001	0.587
			IVW (Q = 413.96, *p* < 0.001)	1.111 (1.060–1.163)	9.35 × 10^−6^		
			Weighted median	1.105 (1.061–1.151)	1.78 × 10^−6^		
			Simple mode	1.059 (0.917–1.222)	0.436		
			Weighted mode	1.141 (0.991–1.314)	0.07		
Depression	HDL-C	90	MR Egger	1.027 (0.837–1.261)	0.796	−0.002	0.337
			IVW (Q = 551.12, *p* < 0.001)	0.932 (0.885–0.981)	0.007		
			Weighted median	0.920 (0.881–0.960)	1.37 × 10^−4^		
			Simple mode	0.865 (0.766–0.977)	0.022		
			Weighted mode	0.911 (0.782–1.062)	0.235		

*IVW* inverse-variance weighted, *MetS* metabolic syndrome, *FBG* fasting blood glucose, *HDL-C* high-density lipoprotein cholesterol.

As to its components, genetic liability to depression was positively related to waist circumference, hypertension, and triglycerides while negatively associated with HDL-C. The ORs with 95% CIs per log_-_odds increment in genetically predicted depression were 1.083 (95% CI: 1.027–1.143; *p* = 0.003) for waist circumference, 1.028 (95% CI: 1.016–1.039; *p* = 1.34 × 10^−6^) for hypertension, 1.111 (95% CI: 1.060–1.163; *p* = 9.35 × 10^−6^) for triglycerides, and 0.932 (95% CI: 0.885–0.981, *p* = 0.007) for HDL-C in the IVW models (Table 2). No significantly association was identified for FBG (OR = 1.010; 95% CI: 0.986–1.034; *p* = 0.423). Those associations were mostly consistent across other models. MR-Egger regression analyses indicated that there was no potential horizontal pleiotropy (all *p* values >0.10) (Table 2). As to heterogeneity, Cochran’s Q test indicated obviously heterogeneities (all *p* values of Cochran’s Q < 0.001), however, most of the funnel plots suggested no evidence of heterogeneity (Supplementary Fig. 2). In addition, the leave-one-out analysis showed that the results were not significantly changed after omitting any single SNP, suggesting the stability of the observed associations (Supplementary Fig. 3).

### The causal effect of MetS and its components on depression

In the reverse MR analysis, after excluding the unavailable SNPs in the summary-level dataset of depression and the palindromic SNPs, we utilized 73 variants for MetS, 30 variants for waist circumference, ten variants for hypertension, 118 variants for FBG, 26 variants for triglycerides, and 60 variants for HDL-C as genetic instruments, respectively. We had high statistical power (>80% power to estimate an OR of 1.20) to assess associations of MetS and its components with depression (Table 1).

As shown in Table 3, the scatter plots (Supplementary Fig. 4), and forest plots (Supplementary Fig. 5), the MR results showed neither MetS nor its five elements were causally related to depression, with ORs close to 1. Egger’s test showed no potential horizontal pleiotropy exists but for the relationship between waist circumference and FBG and risk of depression. Cochran’s Q test and funnel plots (Supplementary Fig. 6) indicated obviously heterogeneities except for the association between hypertension and risk of depression. The leave-one-out analysis also revealed the stability of the results (Supplementary Fig. 7).

**Table 3:** Genetic predicted MetS and its components on the risk of depression in the MR analysis.

Exposure	Outcome	No.SNP	Methods	OR (95% CI)	*p* val	Egger_intercept	*p*-Egger_intercept
MetS	Depression	73	MR Egger	1.022 (0.976–1.071)	0.356	−0.001	0.660
			IVW (Q = 173.68, *p* < 0.001)	1.013 (0.989–1.039)	0.296		
			Weighted median	1.018 (0.993–1.044)	0.157		
			Simple mode	1.018 (0.968–1.070)	0.484		
			Weighted mode	1.012 (0.987–1.039)	0.350		
Waist circumference	Depression	30	MR Egger	0.713 (0.539–0.942)	0.025	0.013	0.004
			IVW (Q = 68.78, *p* < 0.001)	1.099 (0.998–1.209)	0.054		
			Weighted median	1.040 (0.949–1.139)	0.402		
			Simple mode	1.062 (0.929–1.215)	0.383		
			Weighted mode	1.041 (0.927-–1.170)	0.502		
Hypertension	Depression	10	MR Egger	0.868 (0.748–1.007)	0.099	0.009	0.183
			IVW (Q = 7.02, *p* = 0.635)	0.966 (0.930–1.003)	0.071		
			Weighted median	0.948 (0.900–0.998)	0.043		
			Simple mode	0.939 (0.861–1.025)	0.192		
			Weighted mode	0.942 (0.873–1.017)	0.162		
FBG	Depression	118	MR Egger	1.116 (1.034–1.204)	0.006	−0.003	0.006
			IVW (Q = 243.76, *p* < 0.001)	1.022 (0.976–1.070)	0.354		
			Weighted median	1.052 (1.002–1.106)	0.043		
			Simple mode	1.045 (0.962–1.136)	0.300		
			Weighted mode	1.061 (1.017–1.107)	0.007		
Triglycerides	Depression	26	MR Egger	1.031 (0.977–1.087)	0.281	0.000	0.784
			IVW (Q = 34.41, *p* = 0.099)	1.036 (0.999–1.075)	0.059		
			Weighted median	1.027 (0.985–1.071)	0.205		
			Simple mode	1.023 (0.939–1.115)	0.605		
			Weighted mode	1.028 (0.993–1.064)	0.129		
HDL-C	Depression	60	MR Egger	0.994 (0.946–1.045)	0.817	0.001	0.382
			IVW (Q = 106.44, *p* < 0.001)	1.011 (0.978–1.045)	0.517		
			Weighted median	1.010 (0.975–1.047)	0.581		
			Simple mode	1.031 (0.954–1.115)	0.443		
			Weighted mode	1.005 (0.972–1.039)	0.778		

*IVW* inverse-variance weighted, *MetS* metabolic syndrome, *FBG* fasting blood glucose, *HDL-C* high-density lipoprotein cholesterol.

## Discussion

In this bidirectional two-sample MR study, we found that genetically predicted depression was significantly positively associated with risk of MetS, waist circumference, hypertension, and triglycerides while negatively associated with HDL-C. The reverse MR analyses observed no evidence that liability to MetS and its components was associated with depression.

Epidemiological data have been consistently proposed that depression was positively correlated with MetS risk [20, 49, 50]. Several meta-analyses of cohort studies suggested that depression was an independent risk factor for MetS, which agrees with our results [24, 25]. As to the reverse direction, observational studies on the effect of MetS on depression risk were inconclusive. Some cohort studies found that MetS could not independently predict depression risk [51, 52], however, some publications proposed a significant positive association [53, 54]. Our MR analysis showed no evidence supporting a determinate causal effect of MetS on depression, suggesting that the observed association is likely due to confoundings, such as physical activity [55, 56] and diet [57]. In addition, Koponen et al. found that MetS was a risk factor for depression only in females [58]. A prospective cohort study in the French population showed MetS associated with new-onset depressive symptoms in the younger-aged group but not in older-aged subjects [53]. Those data suggested that depression risk in the MetS population might differ depending on the definition of MetS and the participants’ characteristics such as age and sex.

As for the components of MetS, a meta-analysis suggested depression was positively associated with central obesity [59]. In addition, subjects with depression have a significant increase in triglycerides levels and a decrease in HDL-C levels [21, 60]. A cross-sectional study in Iran also showed that depression was associated with elevated blood pressure in the elderly, especially men [61]. Consistent with these evidence, our MR analyses proposed that genetically predicted depression was correlated with high waist circumference (an indicator of abdominal obesity) and triglycerides, lower HDL-C, and hypertension. However, we did not identify a causal relationship between depression and high FBG, although some observational studies pointed to an epidemiological association between them [21, 22, 49]. In contrast, Tang et al. performed an MR analysis using summary-level data from the DIAbetes Genetics Replication And Meta-analysis (DIAGRAM) consortium indicated a positive genetic correlation was present between major depression and type 2 diabetes [62]. This paradox might be due to racial differences, as about 30% of the population in MAGIC were non-European ancestry. For MetS and its components associated with depression, the evidence from epidemiological studies have been inconsistent [51, 63], and no positive correlations were observed in our MR analyses. However, potential horizontal pleiotropy was founded for the associations between waist circumference and FBG and risk of depression. Therefore, further studies were wanted to investigate the causal associations between these factors.

Although the exact mechanisms underlying depression to MetS remain unclear, different hypotheses have been put forward. Firstly, individuals with depression or major depression are prone to have a sedentary lifestyle and a diet rich in fat or carbohydrates [64], leading to an increased risk of developing MetS. Secondly, depression and MetS share common pathophysiological mechanisms in the stress system, such as autonomic nervous system disorder and abnormal activation of the hypothalamus-pituitary-adrenal (HPA) axis [65]. It has been shown that patients with depression present an increased HPA activity [66], and the dysregulation of the HPA axis could affect MetS via influencing abdominal glucose metabolism, fat accumulation, and blood pressure regulation [23]. For example, the activation of the HPA axis resulted in higher levels of cortisol and cortisone [67]. A typical effect of cortisol is to redistribute adipose tissue around the abdominal area, leading to central obesity in the long term [68]. In addition, findings have supported the critical roles of low-grade systemic inflammation, elevated oxidative and nitrosidative stress in depression and MetS [65]. Thirdly, conventional antidepressants may have direct side effects on MetS and its components [69]. For instance, tricyclic antidepressant (TCA) use increased the risk of MetS and weight gain [70]. The usage of serotonin-norepinephrine reuptake inhibitors (SNRIs) has been associated with a higher risk for hypertension [71].

Traditional observational studies suggested a bidirectional relationship between depression and MetS. It might be biased by potential confounding and reverse causality, such as unhealthy lifestyle, antidepressant usage. A key strength of this MR study is that our findings avoided reverse causality and minimized residual confounding. Another strength is that we implemented the most comprehensive dataset for the exposures and the most extensive summary-level data for risk of depression, MetS, and its components, therefore, the power to investigate causal associations was high and the estimated effect magnitudes were more accurately. However, our study also has limitations. First, the functions of the genetic instruments and how they affect the risk factors were not fully understood. Second, we still can’t remove the potential pleiotropy effect that might be concealed by the small number of genetic instruments or small sample size, although the MR-egger intercept showed little horizontal pleiotropism. Third, obvious heterogeneities obtained from individual variants by the Cochran’s Q value were present in the MR analyses. We thus performed the leave-one-out analysis, and the results indicated the stability of the observed associations.

In conclusion, our bidirectional MR study indicated a causal link between depression and MetS (and its components), the reverse direction showed no causal associations. Our findings recommended that the prevention, management, and treatment for depression might be enhanced for MetS and its components prevention.

## Supplementary information

Supplementary materials
