# Supplementary material for: The association between depression and metabolic syndrome and its components: a bidirectional two-sample Mendelian randomization study
Source: Transl Psychiatry. 2021 Dec 13;11:633. doi: 10.1038/s41398-021-01759-z (PMC8668963; doi:10.1038/s41398-021-01759-z)
Supplement: Supplementary file 1 — Supplementary materials [file 41398_2021_1759_MOESM1_ESM.pdf]

Supplementary Information to:

The association between depression and metabolic syndrome and its components: A bidirectional two-sample Mendelian randomization study

Min Zhang M.S.[1], Jing Chen Ph.D.[2], Zhiqun Yin B.S.[3], Lanbing Wang B.S.[4], Lihua Peng Ph.D.[2]*

Affiliations:

1 School of Public Health and Management, Chongqing Medical University, Chongqing 400016, China.

2 Department of Anesthesia and Pain Medicine, The First Affiliated Hospital of Chongqing Medical University, Chongqing 400016, China.

3 Department of Psychiatry and Psychology, No.964 Hospital of People's Liberation Army, Changchun City 130026, Jilin Province, China.

4 Division of medical affairs, The First Affiliated Hospital of Army Military Medical University, Chongqing 400038, China.

Corresponding to:

Lihua Peng, PhD

Department of Anesthesia and Pain Medicine

The First Affiliated Hospital of Chongqing Medical University

No.1 Road Youyi Road, Yuanjiagang Community, Yuzhong District, Chongqing 400016, China

E-mail: plhcqmu@163.com

A list of Supporting Information

Supplementary Table 1 Details of the data sources used in this study. (**Page 3**)

Supplementary Figure 1 The forest plots of the association between genetic predicted depression on MetS and its components in MR analysis. (**Page 4**)

Supplementary Figure 2 The funnel plots of the association between genetic predicted depression on MetS and its components in MR analysis. (**Page 5**)

Supplementary Figure 3 The leave-one-out analysis of the association between genetic predicted depression on MetS and its components in MR analysis. (**Page 6**)

Supplementary Figure 4 The scatter plots of the association between genetic predicted MetS and its components on depression in MR analysis. (**Page 7**)

Supplementary Figure 5 The forest plots of the association between genetic predicted MetS and its components on depression in MR analysis. (**Page 8**)

Supplementary Figure 6 The funnel plots of the association between genetic predicted MetS and its components on depression in MR analysis. (**Page 9**)

Supplementary Figure 7 The leave-one-out analysis of the association between genetic predicted MetS and its components on depression in MR analysis. (**Page 10**)

Supplementary Table 1 Details of the data sources used in this study.

Factors	Exposure				Outcome		
	PMID	Samples	Consortium or cohorts	No. of SNPs	Consortium	Samples	Source
Depression	30718901	2113907	23andMe, Psychiatric Genomics Consortium (PGC) and UK Biobank	97	UK Biobank and PGC	2113907	https://datashare.is.ed.ac.uk/handle/10283/3203
MetS	31589552	291107	a GWAS in UK Biobank	79	UK Biobank	291107	https://www.ebi.ac.uk/gwas/downloads/summary-statistics
WC	25673412	224459	the Genetic Investigation of ANthropometric Traits (GIANT) consortium	31	MRC-IEU UK Biobank	462166	https://gwas.mrcieu.ac.uk/
Hypertension	21909115	203006	a GWAS of 29 studies	12	MRC-IEU UK Biobank	463010	https://gwas.mrcieu.ac.uk/
FBG	34059833	281416	the Meta-Analyses of Glucose and Insulin-related traits Consortium (MAGIC)	134	MAGIC	281416	https://magicinvestigators.org/
Triglycerides	24097068	188577	Global Lipids Genetics Consortium (GLGC)	28	UK Biobank	441016	https://gwas.mrcieu.ac.uk/
HDL-C	24097068	188577	Global Lipids Genetics Consortium (GLGC)	60	UK Biobank	403943	https://gwas.mrcieu.ac.uk/

MetS: metabolic syndrome; WC: waist circumference; FBG: fasting blood glucose; HDL-C: high-density lipoprotein cholesterol

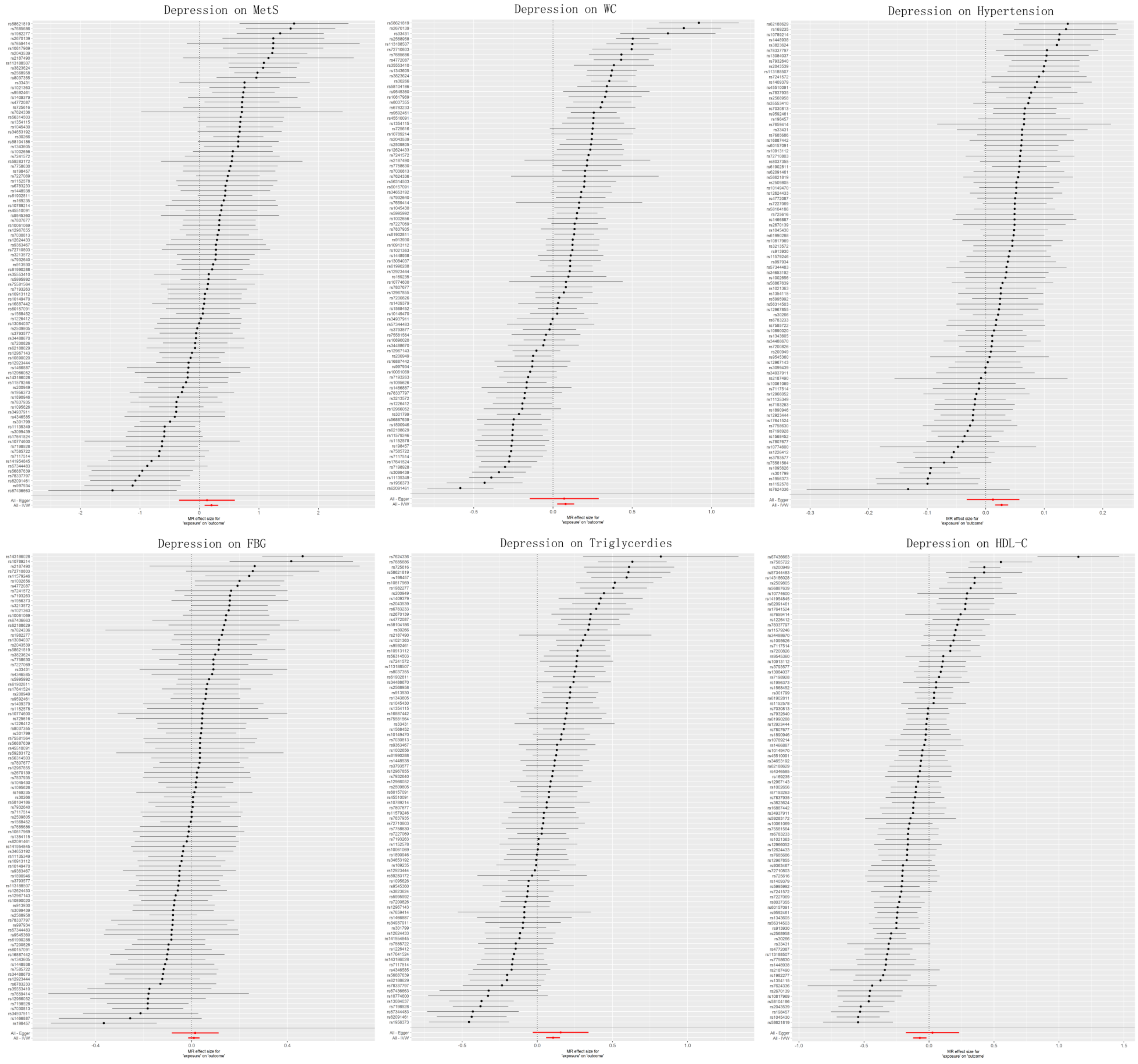

Supplementary Figure 1 The forest plots of the association between genetic predicted depression on MetS and its components in MR analysis. MetS: metabolic syndrome; WC: waist circumference; FBG: fasting blood glucose; HDL-C: high-density lipoprotein cholesterol.

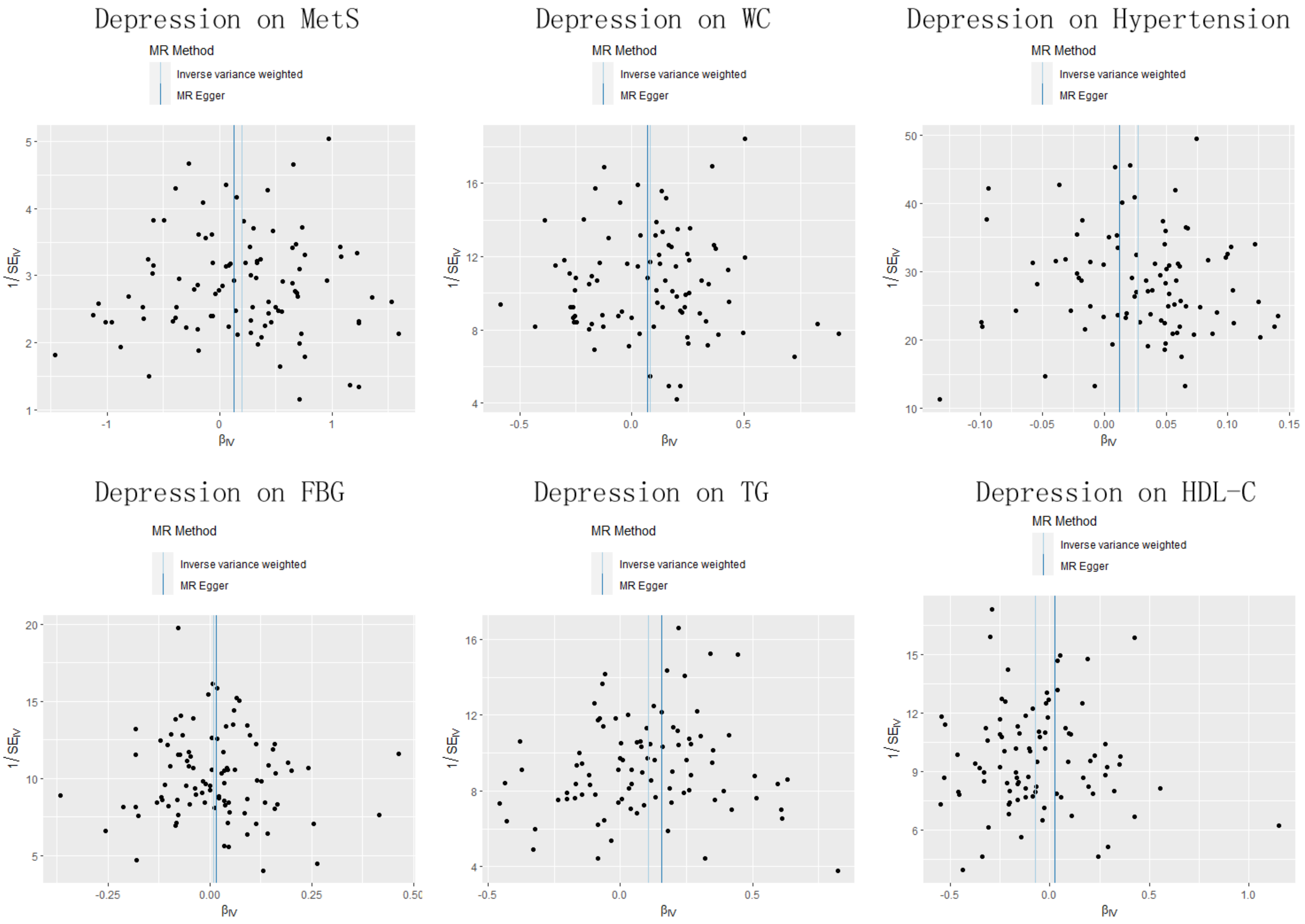

Supplementary Figure 2 The funnel plots of the association between genetic predicted depression on MetS and its components in MR analysis. MetS: metabolic syndrome; WC: waist circumference; FBG: fasting blood glucose; TG: triglycerides; HDL-C: high-density lipoprotein cholesterol.

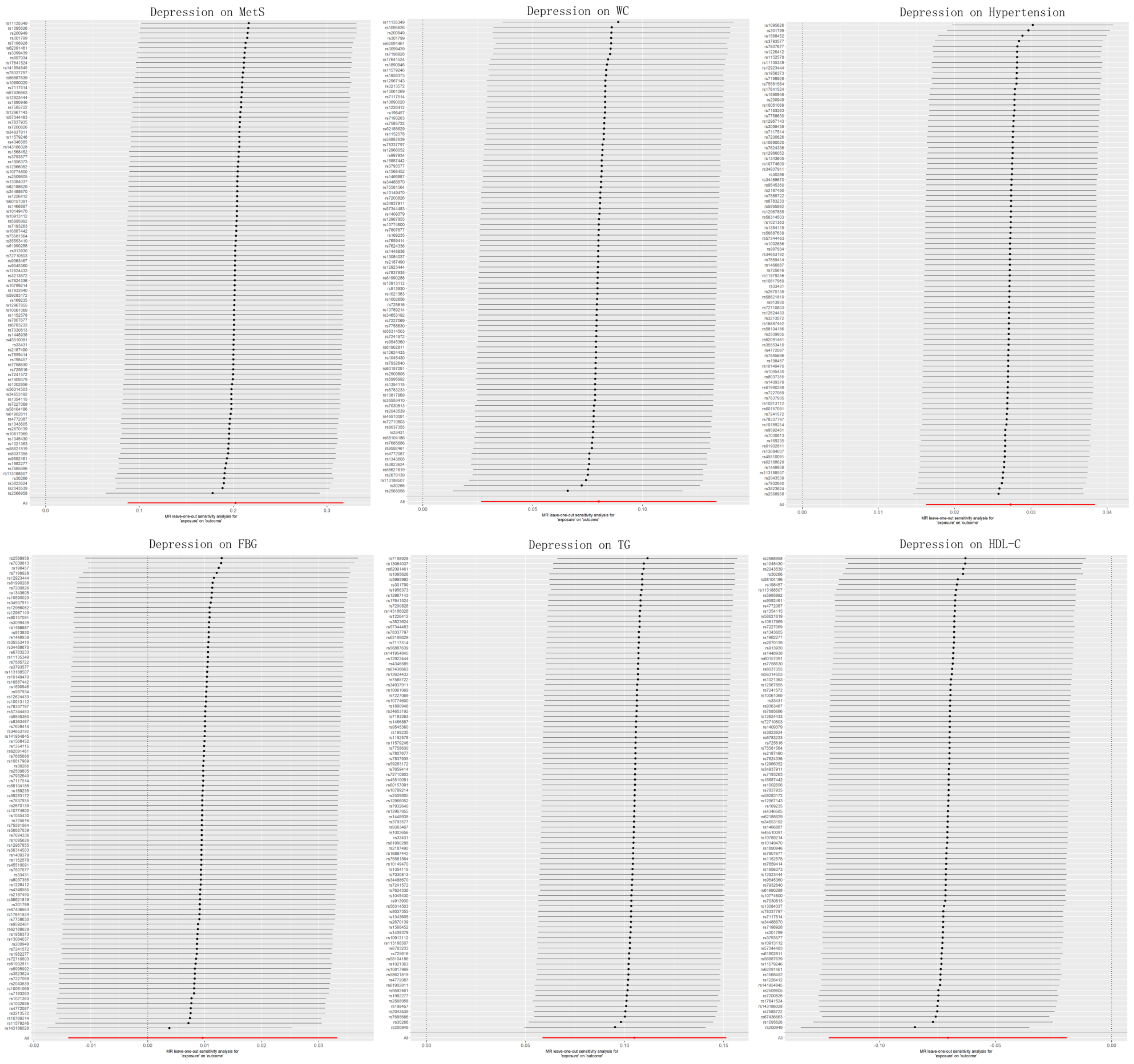

Supplementary Figure 3 The leave-one-out analysis of the association between genetic predicted depression on MetS and its components in MR analysis. MetS: metabolic syndrome; WC: waist circumference; FBG: fasting blood glucose; TG: triglycerides; HDL-C: high-density lipoprotein cholesterol.

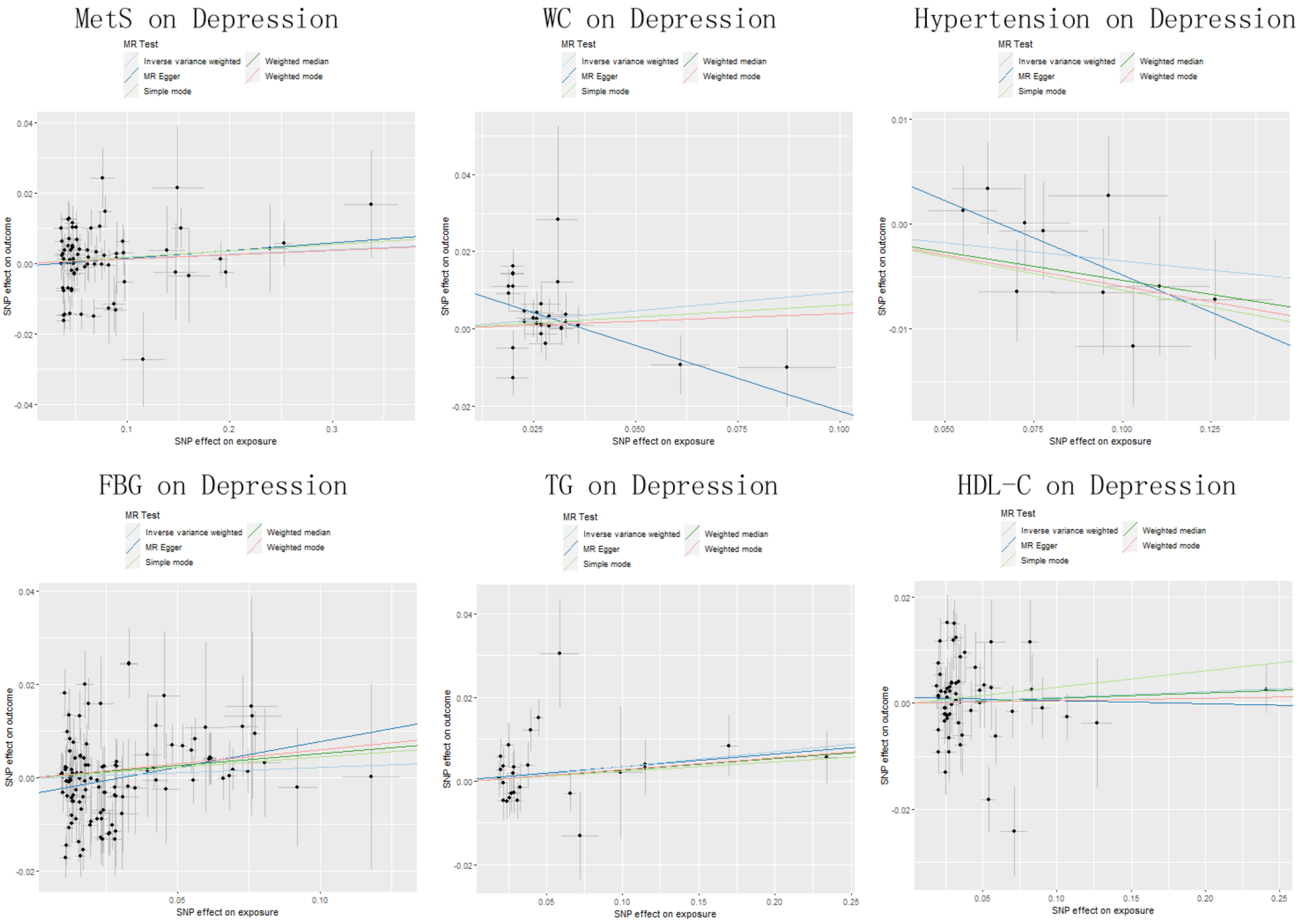

Supplementary Figure 4 The scatter plots of the association between genetic predicted MetS and its components on depression in MR analysis. MetS: metabolic syndrome; WC: waist circumference; FBG: fasting blood glucose; TG: triglycerides; HDL-C: high-density lipoprotein cholesterol.

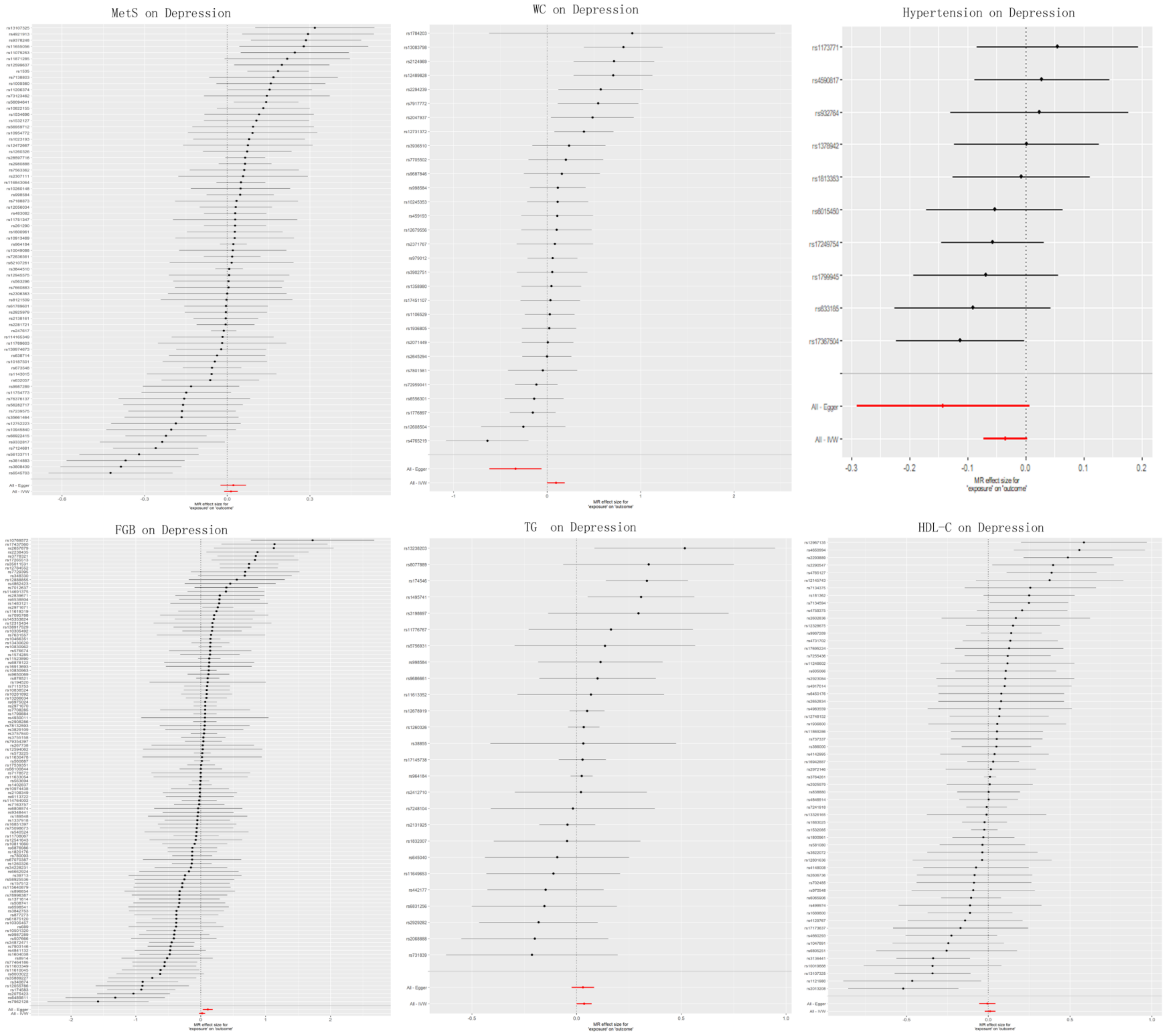

Supplementary Figure 5 The forest plots of the association between genetic predicted MetS and its components on depression in MR analysis. MetS: metabolic syndrome; WC: waist circumference; FBG: fasting blood glucose; TG: triglycerides; HDL-C: high-density lipoprotein cholesterol.

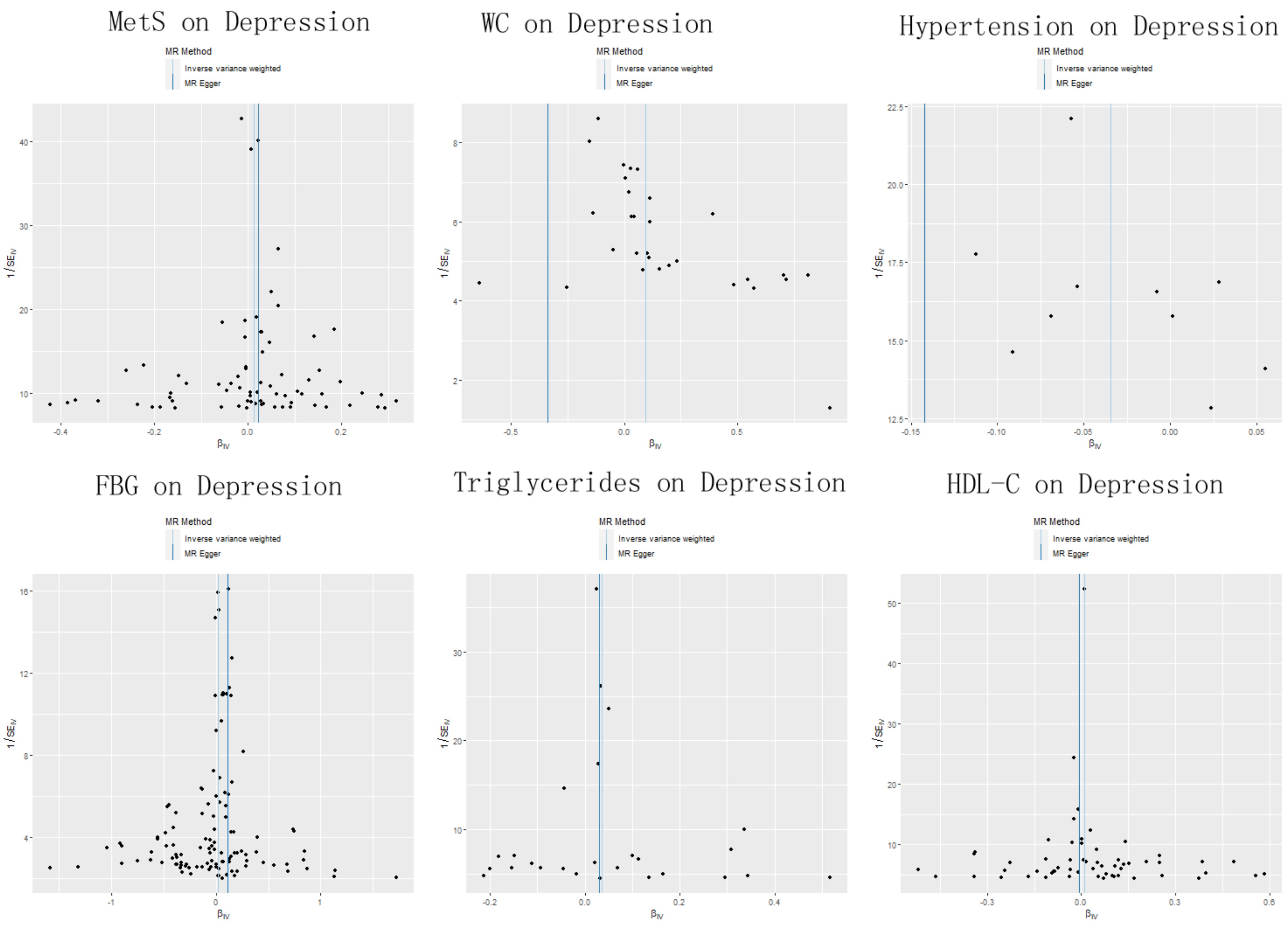

Supplementary Figure 6 The funnel plots of the association between genetic predicted MetS and its components on depression in MR analysis. MetS: metabolic syndrome; WC: waist circumference; FBG: fasting blood glucose; HDL-C: high-density lipoprotein cholesterol.

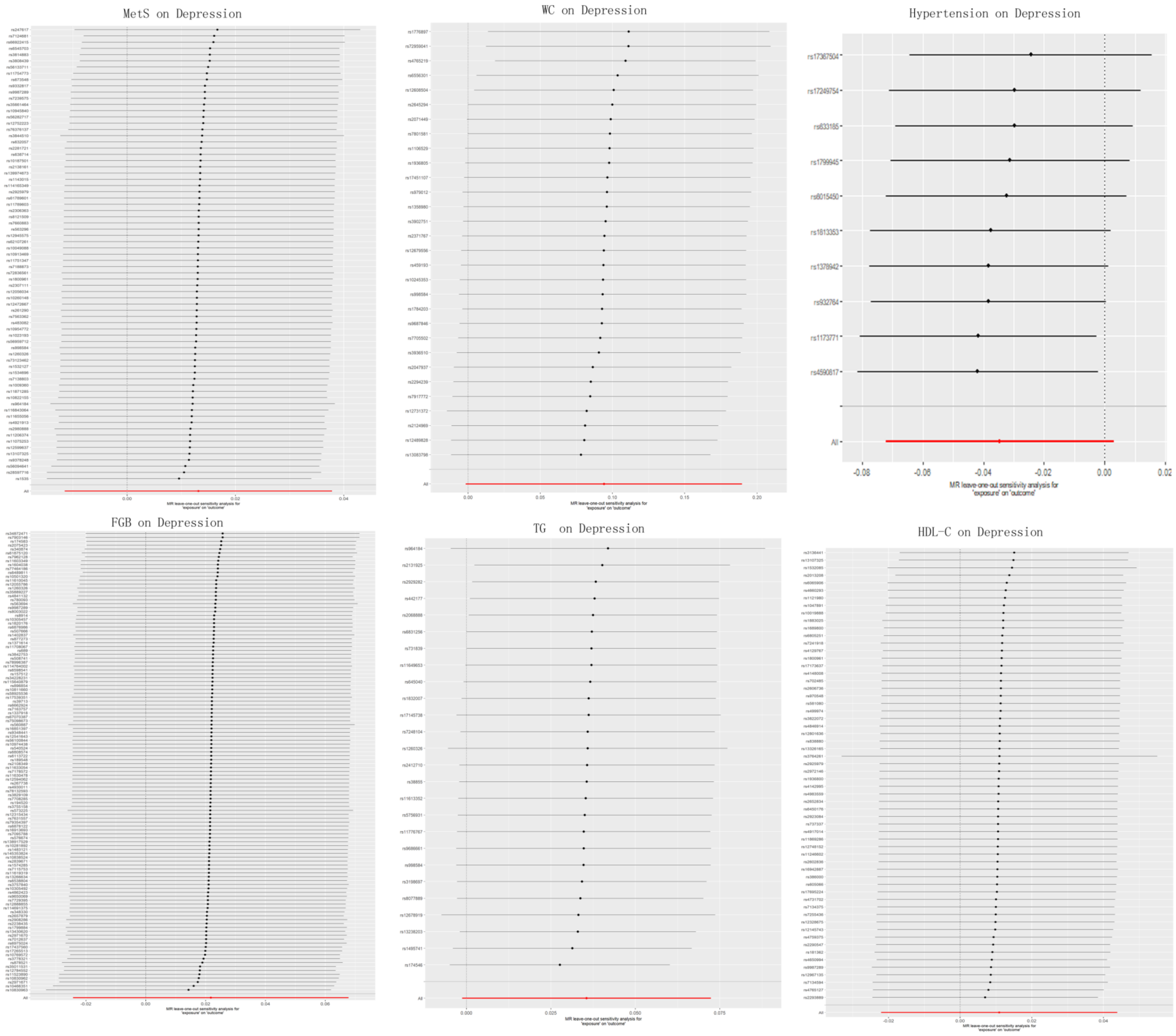

Supplementary Figure 7 The leave-one-out analysis of the association between genetic predicted MetS and its components on depression in MR analysis. MetS: metabolic syndrome; WC: waist circumference; FBG: fasting blood glucose; TG: triglycerides; HDL-C: high-density lipoprotein cholesterol.
